# An integrative head–heart–hands model of moral education: evidence from Chinese higher education

**DOI:** 10.3389/fpsyg.2026.1762483

**Published:** 2026-02-23

**Authors:** Meng Wang, Norzihani Saharuddin, Maizura Yasin, Xu Chen

**Affiliations:** 1 Faculty of Educational Studies, University Putra Malaysia, Serdang, Selangor, Malaysia; 2 School of Marxism, Henan Institute of Science and Technology, Xinxiang, Henan, China

**Keywords:** character education, head–heart–hands (3H), holistic education, moral affect, moral cognition, moral education, moral behavior, Chinese higher education

## Abstract

**Introduction:**

Traditional moral education often treats cognition, affect, and behavior as separate targets, limiting integration of knowing, feeling, and doing. This study examines a head–heart–hands (3H) moral education model and its implementation in a mandatory Chinese university course.

**Methods:**

We conducted a 19-week 3H-informed intervention in three parallel classes (total enrolment = 175) of an “Ideology, Morality and Rule of Law” course. The design was an action-research-informed qualitative case study. Data included classroom observations, student reflective journals, and semi-structured interviews with 12 focal students.

**Results:**

Thematic analysis found that students reported deeper contextualized moral reasoning and critical reflection (Head), stronger value-related resonance and empathy (Heart), and more sustained engagement in action-oriented practices (Hands). Across the semester, reasoning, affect, and action appeared to form a dynamic, recursive cycle of moral growth.

**Discussion:**

These exploratory findings suggest that aligning instructional activities with head, heart, and hands can support integrated moral learning. We discuss implications and boundary conditions for applying the 3H model in compulsory university moral education.

## 1 Introduction

In an era of profound social and technological transformation, higher education faces an urgent mission: not only to develop students’ knowledge, but also to cultivate their character, virtues, and sense of social responsibility (Arthur, 2024; Godonoga and Sporn, 2023). This mission arises against the backdrop of growing global interconnectedness and the spread of digital media, which have fundamentally reshaped interpersonal interaction and value ecologies and created unprecedented ethical challenges (Lavenne-Collot et al., 2022; UNESCO, 2021). In response, UNESCO has called for a “renewal of humanity’s moral compass,” underscoring that education needs to move beyond the transmission of information to foster students’ socio-emotional competencies and ethical agency (UNESCO, 2012).

Despite this broad consensus, a persistent gap remains between the stated aims and everyday practices of moral education. Mainstream approaches in higher education still place disproportionate emphasis on cognitive reasoning while paying insufficient attention to emotional engagement and embodied action (Rest and Narvaez, 2014). This imbalance can produce a structural separation among moral cognition, moral affect, and moral behavior, undermining the internalization of values and their translation into sustained conduct (Narvaez, 2006, 2021). For instance, analysis of PISA 2018 data suggests that while more than half of students endorse environmentally enthusiastic values and attitudes, only about one-fifth report being actively involved in environmental actions (OECD, 2022). A narrowly rationalist orientation may therefore produce individuals who can articulate ethical principles but lack empathy and practical capacities to enact these principles in complex real-world contexts (Kristjánsson, 2025; Sandel, 2020). In countries such as China, this challenge is especially salient, since compulsory moral education courses, such as “Ideology, Morality and Rule of Law” (思想道德与法治), are often dominated by lecture-based instruction, leaving students with limited opportunities for experiential learning and deep value internalization (Eryong and Li, 2021; Liu et al., 2023).

Scholars have examined different dimensions of moral development in considerable depth. Foundational work has established stage-based theories of moral reasoning (Kohlberg, 1984; Rest, 1992), highlighted the centrality of care ethics and empathy (Gilligan, 1982; Hoffman, 1996; Noddings, 1984), and underscored the importance of community-engaged practice (Bandura, 2011; Colby and Damon, 1992; Dewey, 1986; Eisenberg et al., 2016). In response to the limitations of single-focus approaches, integrative paradigms have emerged. Neo-Aristotelian character education, for example, conceptualizes virtue as a unified disposition that integrates judgment, emotion, and habit (Arthur et al., 2022; Carr, 2024; Kristjánsson, 2019). Lickona’s (1991) character education emphasizes the integration of “knowing, feeling, and doing”, and Integrative Ethical Education (IEE) advocates for building an ecosystem that supports the development of ethical skills (Narvaez, 2006). Despite these advances, a critical gap remains: there are few practical models that can dynamically and systematically integrate cognition, affect, and action within a classroom-tested framework. In addition, there is a notable lack of robust empirical research from non-Western contexts, particularly from settings such as China with distinct cultural and institutional characteristics, which constrains meaningful cross-cultural comparison and mutual learning.

To address these theoretical and practical challenges, the present study introduces and empirically examines a head–heart–hands (3H) process model of moral education. Building on established tripartite ideas (e.g., knowing–feeling–doing), the model specifies a recursive classroom mechanism in which action-oriented tasks generate feedback for subsequent reflection and value appraisal, and it operationalises this mechanism into activity sequences that can be examined with multi-source qualitative evidence. Related 3H framings have appeared in sustainability and holistic education, but systematic implementation and qualitative examination within a compulsory Chinese university moral education course remains limited.

Against this backdrop, we integrated the 3H moral education model into a core compulsory course, “Ideology, Morality and Rule of Law,” at a public university in China. Drawing on a 19-week action research and qualitative case study design, we examined how the model was implemented in classroom practice and how it influenced students’ development. The study was guided by the following research questions:

1. How does the 3H intervention deepen students’ moral cognition?
2. How does it foster students’ moral affect?
3. How does it support changes and sustained engagement in students’ moral behavior?
4. Within the 3H framework, how do moral cognition, moral affect, and moral behavior interact in a dynamic, spiraling process?

## 2 Literature review: from fragmented moral education to holistic integration

The core aim of moral education is to cultivate persons with a coherent moral character that integrates moral cognition, moral affect, and the capacity for moral practice (Lickona, 2013; Saharuddin et al., 2021). Since the twentieth century, several influential theoretical approaches have emerged, each illuminating one important dimension of moral development, yet often doing so in a fragmented way due to their narrow focus. This section first critically reviews three major approaches—cognitive-rational, care-oriented, and participatory-constructivist—before synthesizing the growing calls for holistic integration. It then traces the evolution of the head, heart and hands (3H) framework, from its philosophical roots in holistic education to its contemporary interpretations and finally introduces our process-oriented 3H account as a response to the identified theoretical gaps.

### 2.1 Major approaches in moral education: strengths and limitations

#### 2.1.1 Cognitive-rational approaches

Cognitive-rational approaches are rooted in Piaget’s (1932) theory of cognitive development and were later elaborated into Kohlberg’s (1976, 1984) stage theory of moral reasoning. From this perspective, moral growth is achieved through a series of hierarchically ordered cognitive stages. Moral development is promoted by structured engagement with moral dilemmas, which is expected to stimulate cognitive conflict and perspective taking. Initiatives such as the Just Community–inspired work at the Haverford (often translated as “Farrington”) project (Kohlberg and Power, 1981) and the rational-construction methods of Shaver and Strong (1982) further emphasize structured dialogue and rational judgment to help adolescents autonomously construct and resolve value conflicts.

Although this tradition has provided a valuable account of the evolution of moral judgment, its main limitation lies in reducing morality to justice-based reasoning and largely overlooking the roles of emotion, virtue, and embodied action (Gibbs, 2019; Nucci, 2001). Within the 3H model, this approach offers a solid foundation for the “head” by clarifying how moral cognition develops. However, it pays insufficient attention to the motivational dynamics of the “heart” and the habitual dimensions of the “hands,” which may result in individuals who can think abstractly about ethical issues but lack corresponding empathy or stable practices.

#### 2.1.2 Care-oriented approaches

In response to rationalist emphases, care-oriented approaches, pioneered by Gilligan (1982) and developed by Noddings (1984, 2002), highlight care and relational ethics. Gilligan’s landmark work challenged Kohlberg’s justice-oriented hierarchy by arguing for a distinct moral voice organized around responsibility, care, and the avoidance of harm. Noddings (2002) extended this line of thought by proposing an “ethic of care” that shifts the focus of moral education from abstract principles of justice to concrete relationships and affective resonance.

Pedagogically, McPhail’s (1972) “Consideration Approach” seeks to cultivate interpersonal empathy by creating a classroom climate characterized by care and mutual respect, while Noddings’s “Caring Community Approach” aims to build a culture of reciprocity and mutual support. These approaches have been effective in fostering empathy, compassion, and harmonious interpersonal relationships. However, critics point out that they may insufficiently specify the role of cognitive and deliberative processes. Without complementary principles of justice and critical reflection, there is a risk of parochialism or relativism (Baker et al., 1997; Bergman, 2004; Jackson, 2025).

#### 2.1.3 Participatory-constructivist approaches

Participatory-constructivist approaches combine insights from pragmatism and social constructivism, arguing that moral understanding emerges through democratic dialogue, social action, and collaborative inquiry. Drawing on Dewey’s emphasis on “learning by doing” in social contexts in Experience and Education and Bandura’s (1991) social learning theory, these approaches view moral behavior as shaped through modeling, observation, and self-regulation in interaction with others.

Curricular innovations such as Stenhouse’s (1968) Humanitarian Curriculum Project link moral learning with real-world humanitarian issues. Kohlberg’s (2013) Just Community Approach creates school-based micro-democracies in which student’s experience collective rule making and fair procedures. Collaborative approaches further highlight the construction of moral meaning through negotiated dialogue and consideration of diverse perspectives (Gartland and Field, 2004; Johnston and Buzzelli, 2002; Kotsonis, 2022).

Although participatory approaches are effective in situating morality in concrete contexts and cultivating civic agency, they may drift toward relativism if not grounded in robust cognitive and affective development (Oser, 2014; Oser et al., 2008). Without an articulated account of how moral cognition, moral affect, and moral behavior interact, participation alone may not guarantee coherent moral growth.

**Figure 1:**
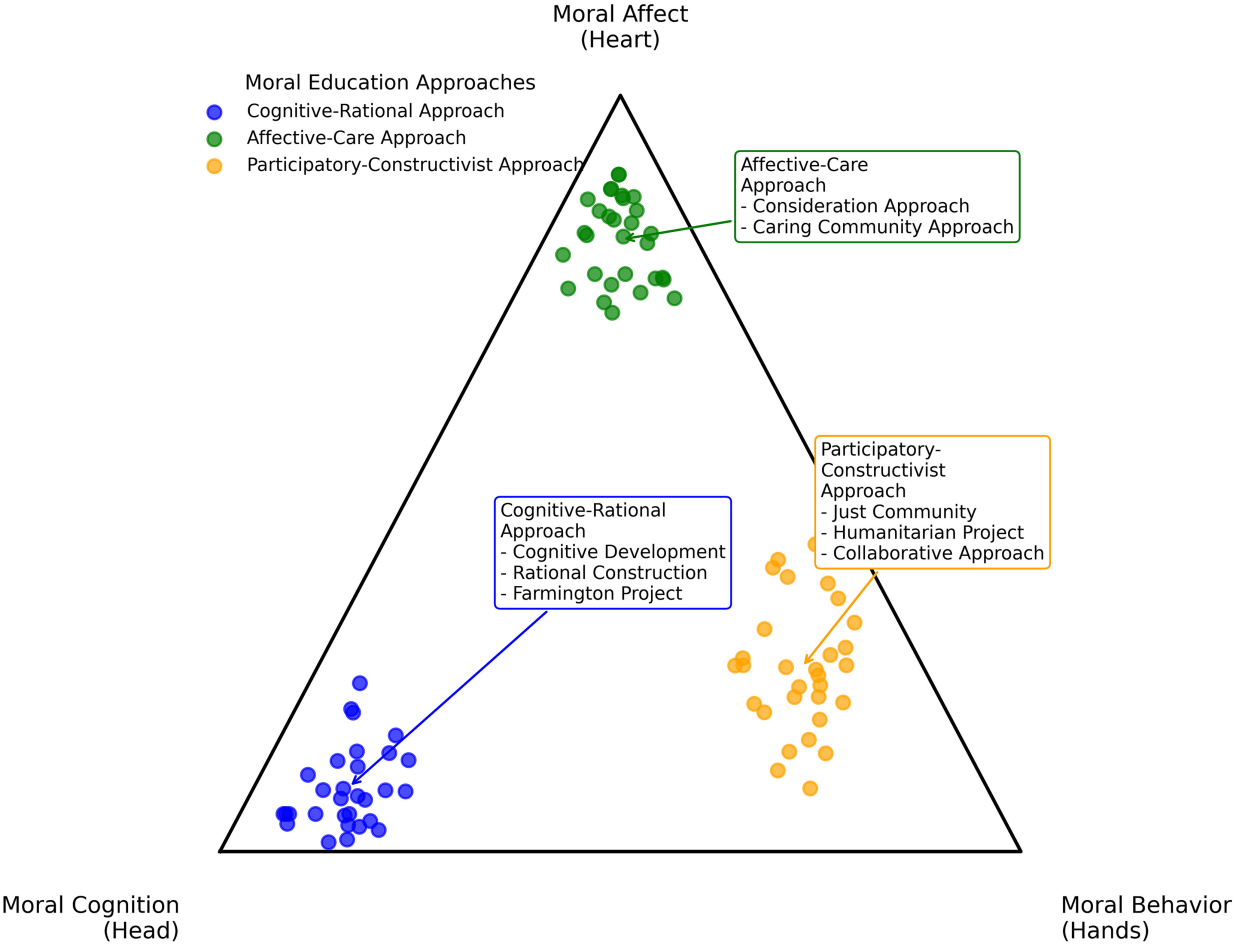
Characteristics of moral education approaches. This ternary diagram positions the three approaches within a moral cognition–moral affect–moral behavior space, indicating their differing emphases. The pedagogical methods are not fixed in their location; their positions may shift depending on specific practices.

Figure 1 visually illustrates how the three approaches are distributed across the dimensions of cognition, affect, and action. Like the proverbial blind men and the elephant, each approach grasps an important component of moral education, yet none captures the whole. They share three major limitations: (1) each tends to overemphasize one dimension (cognition, affect, or behavior) while neglecting the others; (2) there is often a persistent gap between theory and practice, with insufficient linkage among moral knowing, moral feeling, and moral doing; and (3) they pay inadequate attention to the dynamic processes through which these dimensions interact to form holistic moral development. In the face of contemporary moral challenges, no single approach appears sufficient (Narvaez, 2006). From a neo-Aristotelian standpoint, the perspective of practical wisdom and flourishing underscores that education should not merely teach “individual virtues,” but should cultivate students’ capacity to deliberate and judge in complex situations (McLoughlin et al., 2025). Developing a framework that can integrate these dimensions and form a dynamic cycle has thus become an urgent task for the advancement of moral education theory (Gazibara, 2013; Ma, 2009; Sanger and Osguthorpe, 2005).

### 2.2 Calls for holistic integration and the legacy of the 3H model

The need for integration is not new. The tripartite ideal of “head, heart and hands” can be traced back to the eighteenth-century Swiss educational reformer Pestalozzi, who advocated balanced development of intellectual, moral, and physical capacities. This holistic paradigm has gained renewed recognition in recent decades. “Holistic learning” frameworks emphasize that through authentic, context-rich experiences, students can develop their cognitive, emotional, and volitional capacities in an integrated way (Miller et al., 2019).

Orr (1991), in his work on ecological literacy, explicitly revived the 3H idea, reconceptualizing reflective knowledge, affective engagement, and participatory practice as a unified cycle. Sipos et al. (2008) applied this framework to education for sustainability, explicitly linking the head with critical thinking, the Heart with empathy and care, and the Hands with sustainable practices. At the same time, Dewey’s principles of experiential learning—that knowledge emerges from the active reconstruction of experience through reflection—provide an essential theoretical bridge that connects action (hands) with understanding (head) and value (heart).

In contemporary moral philosophy and character education, Lickona (1991) proposed a tripartite structure of “moral knowing, moral feeling, and moral action,” directly targeting the simultaneous cultivation of moral cognition, moral affect, and moral behavior. Narvaez’s (2006) Integrative Ethical Education (IEE) model attempts to build systematic linkages among cognition, affect, and social action through caring teacher–student relationships and communities of practice that support the formation of moral habits. However, although the IEE model achieves multidimensional integration at a theoretical level, its classroom-level applicability and cross-cultural adaptability remain constrained (Narvaez, 2021).

Similarly, Neo-Aristotelian character education returns to the tradition of virtue cultivation and emphasizes the unity of knowing, desiring, and doing the good (Arthur and Kristjánsson, 2022; Carr and Steutel, 2005; Kristjánsson, 2019). This vision aligns conceptually with head–heart–hands, yet many existing tripartite frameworks remain largely descriptive. Our use of the 3H model places particular emphasis on specifying a recursive learning process and on translating that process into teachable and observable classroom practices. In this sense, the contribution is not the tripartite structure itself, but the process account and its pedagogical operationalization.

International practices have further demonstrated the potential of integrative models. Singapore’s Character and Citizenship Education curriculum, for example, systematically brings together cognition, affect, and action through narratives, role play, and service learning (Ministry of Education, Singapore, 2021a, 2021b, 2021c). In Latvia, the e-TAP project found that when 56% of activities and more than 80% of modules achieved an organic integration of head, heart, and hands, moral education had deeper impacts on students (Surikova and Sidorova, 2023). Climate education projects in Israel have also shown that 3H-inspired approaches help students acquire knowledge (head), maintain positive emotions and concern (heart), and translate these into sustained willingness to act (hands) (Gal, 2024).

Despite these encouraging trends at the macro level, significant gaps remain. As Miseliunaite et al. (2022) note, empirical research that explicitly examines holistic approaches within moral education is still limited and is often subsumed under broader agendas such as social–emotional learning or spiritual growth. There is a lack of validated, micro-level frameworks that can systematically connect moral cognition, empathy, and ethical behavior within a dynamic process model. The present study aims to address this gap.

### 2.3 The head, heart and hands (3H) model: an integrative and recursive framework

Building on the above critical synthesis, this study develops a holistic moral education model of head, heart and hands (3H). The model is grounded in three interrelated theoretical layers:

(a) Pestalozzi’s historical ideal of educating the head, heart, and hands as a whole person;
(b) Contemporary character education and virtue ethics, including Lickona’s character education, Narvaez’s Integrative Ethical Education, and neo-Aristotelian virtue theory;
(c) Moral psychology and educational theory, including Kohlberg’s cognitive-developmental theory, Noddings’s ethic of care, and Dewey’s pragmatist philosophy of experience.

These theoretical layers are further interpreted through the cultural lens of Chinese moral education and the Confucian ideal of the unity of knowing and doing (知行合一).

**Figure 2:**
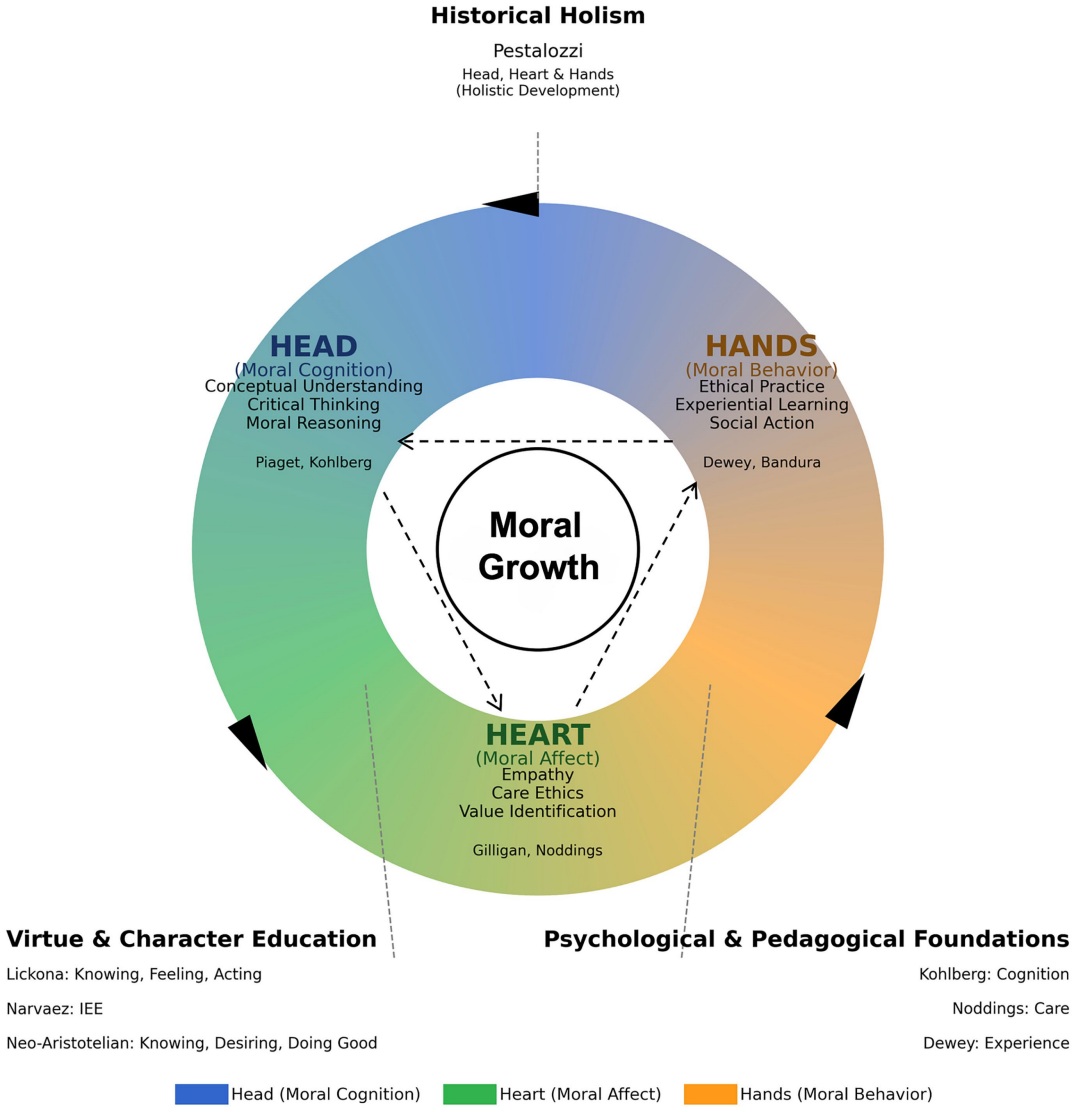
The head, heart and hands (3H) model for moral education (developed by the authors based on Dewey, 1986; Kohlberg, 1976; Noddings, 2013; Pestalozzi, and subsequent research).

Historically, the 3H model echoes Pestalozzi’s call for balanced cultivation of head, heart, and hands. In the present framework, these are specified as moral cognition (head), moral affect (heart), and moral behavior (hands). Figure 2 summarises our process account as a recursive sequence intended to guide both pedagogical design and qualitative interpretation.

In this process account, learning proceeds through repeated unit cycles: conceptual clarification and moral deliberation (head) is linked to affective resonance and value identification (heart), which is then connected to action-oriented participation (hands). Crucially, Hands is not treated as an endpoint. Action episodes and social feedback are brought back through structured reflection, which reshapes subsequent reasoning and emotional appraisal (hands → head) and allows the cycle to deepen over time rather than unfold as a one-off progression.

This process account complements knowing–feeling–doing (Lickona, 2013) and character cultivation perspectives (Arthur et al., 2022) by specifying how the three domains can be intentionally linked within classroom design, and by treating action as epistemically and affectively generative rather than merely an outcome. The neo-Aristotelian lens further underscores that the recursive cycle is normatively oriented toward moral growth and flourishing, rather than toward compliance or efficient functioning alone.

Finally, the framework is designed to be culturally adaptable. In the Chinese context, it resonates deeply with the Confucian ideal of the unity of knowledge and action (Fengyan, 2004) and with the national educational agenda of “fostering virtue through education and promoting students’ all-round development” (Communist Party of China, 2022). The tripartite structure of head, heart and hands offers a flexible yet systematic framework for integrating local values, indigenous narratives, and policy priorities into moral education.

At the same time, integrationist approaches are not without critique. Scholars have cautioned that aligning cognition, affect, and action can be normatively and methodologically challenging: strong affective appeals may risk emotional manipulation or moralization, and enacted “moral behavior” in educational settings may reflect compliance, impression management, or situational pressure rather than stable moral commitment. Process models also risk over-smoothing variability by presenting learning as coherent cycles, when in practice students may experience discontinuities, resistance, or ambivalence. These tensions inform our analytic stance in the present study: we treat the 3H cycle as a sensitizing framework for interpreting patterns in qualitative data, while remaining attentive to variation, boundary conditions, and context-specific constraints.

In this study, we operationalized the 3H model in a public university in China. The intervention deliberately combined stories, role play, reflective journals, and community-oriented tasks to activate and connect the three dimensions in culturally meaningful ways. The subsequent chapters explain in detail how this framework guided the design, implementation, and evaluation of the intervention.

## 3 Methods

### 3.1 Study purpose and context

Guided by an action research orientation (Lewin, 1946) and a qualitative case study approach (Creswell and Poth, 2016), this study implemented and examined a 19-week moral education intervention informed by the head–heart–hands (3H) framework. The study addressed four research questions on students’ moral cognition, moral affect, moral behavior, and their dynamic interrelationships.

The intervention was conducted at a comprehensive university in central China, selected purposively because it has promoted moral education reform since 2024 and was willing to embed a 3H-informed approach within the compulsory course “Ideology, Morality and Rule of Law”. In this context, teaching has typically been lecture-dominant with limited experiential learning opportunities (Eryong and Li, 2021; Zhang, 2022).

### 3.2 The 3H intervention

The intervention systematically embedded the 3H framework into “Ideology, Morality and Rule of Law”, a compulsory undergraduate course prescribed within the national curriculum (Ministry of Education of the People’s Republic of China, 2021). Without altering mandated syllabus themes, we used 3H as a design principle to support iterative cycles of reasoning, resonance, and action across moral cognition (head), moral affect (heart), and moral behavior (hands).

#### 3.2.1 Teacher collaboration and co-construction

A research–teaching collaborative community was established. Before the semester, the research team and course instructors conducted three co-design workshops to align 3H principles with course units. The team then co-developed 2–3-week “instructional unit packages” containing case materials, activity guidelines, and reflective tools with explicit head/heart/hands aims (see Supplementary Table S1 for the semester-wide unit-package map). Additionally, during the semester, the team met approximately biweekly to review student feedback and observation notes and refine subsequent units, consistent with action research cycles (Kemmis et al., 2013).

#### 3.2.2 Course structure and activity design: an integrative example

A typical 2–3-week unit followed the head–heart–hands (3H) sequence and incorporated structured reflection that connected action experiences back to subsequent reasoning (returning to head). Table 1 provides an overview of core interactive exercises across the semester, Supplementary Table S1 provides a semester-wide overview of the 2–3-week unit packages across the 19-week intervention, mapping each unit’s core theme/value to its primary head/heart/hands learning activities, while Supplementary Table S2 presents an exemplar unit showing sequencing, key activity nodes, and where learning evidence was generated.

**Table 1:** Core interactive exercises implemented across the semester-long 3H course.

Core interactive exercise (all students)	Primary 3H focus (dominant target)	Brief implementation (how it was done)	Primary evidence source(s) used in thematic analysis
Concept instruction with guided questioning	Head (moral cognition)	Short theoretical input on key concepts (e.g., legal awareness, Socialist Core Values) followed by instructor questioning to help students structure key points and clarify misunderstandings.	OBS; RJ
Case analysis and moral dilemma discussion	Head (moral cognition)	Small-group analysis of real or simulated cases and dilemmas, focusing on reasons, consequences, and possible solutions; groups report back.	OBS; RJ
Video/film-based moral episode analysis	Heart (moral affect)	Viewing moral-related clips (e.g., peer role models, helping others), followed by guided prompts for emotional and value-oriented interpretation.	OBS; RJ
Storytelling and personal narrative sharing	Heart (moral affect)	Students connect topics to personal or family experiences and share narratives to trigger peer empathy and value resonance.	OBS; RJ; SI
Role-play embedded in group projects (including micro-film)	Heart (moral affect) as primary; also supports head and hands	Groups self-organize and self-assign roles (e.g., stakeholder roles or production roles such as director/editor). All students participate, although role experiences differ across individuals.	OBS; RJ; SI
Social investigation tasks (campus/community)	Hands (moral behavior)	Students conduct field investigation or surveys linked to unit themes; results are summarized and reflected on.	RJ; SI; OBS
Service learning/volunteering activities	Hands (moral behavior)	Participation in campus volunteering or community service with structured reflection linking action back to course concepts.	RJ; SI; OBS
Integrity pledge and integrity charter (Integrity unit)	Hands (moral behavior)	Class-based pledge ceremony and signing integrity commitment, followed by reflection on feasibility and personal responsibility.	RJ; OBS
Community legal literacy outreach (rule-of-law unit)	Hands (moral behavior)	Students deliver legal literacy communication in communities and write practice reports linking experience to course concepts.	RJ; SI; OBS
Peer review and peer assessment	Integration (hands → head)	Groups critique each other’s proposals/products (feasibility, values alignment, evidence quality) and revise accordingly.	OBS; RJ
Structured reflective journals (continuous)	Integration (head–heart–hands)	Regular prompts require linking what was understood (head), felt (heart), and enacted/planned (hands).	RJ; SI
Multi-source formative assessment (quizzes, presentations, observation, self-evaluation)	Evaluation across 3H	Knowledge quizzes and in-class questioning; presentation scoring for reasoning and value articulation; observation logs; self-evaluation.	OBS; RJ

The “Primary 3H focus” column indicates the dominant instructional target; several exercises were designed to support multiple dimensions (head, heart, and hands) through iterative linkage across sessions. OBS, classroom observation notes; RJ, reflective journals; SI, semi-structured interviews (focal cases only).

It is important to note that students’ experiences were not identical across activities. For example, in a moral micro-film project on campus bullying, groups self-organized and allocated both situational roles (e.g., victim, perpetrator, and bystander) and task roles (e.g., director, scriptwriter, and editor). This design allowed multiple participation pathways into moral perspective and affective engagement, including embodied role enactment and reflective meaning-making through production, discussion, and peer collaboration. We do not treat technical skills as moral competence per se; rather, we examine how such competencies can function as action-enabling capacities within the Hands dimension, supporting communicable and collaborative enactments of moral commitments.

### 3.3 Participants

The study took place in a public university in Henan Province, China. The first-year cohort included 2,913 students across 145 discipline-based small classes, administratively organized into 50 larger classes. The research team invited these administrative classes to participate as intact classes in a 3H-informed learning experience and to allow the use of anonymized classroom learning materials and participation records for research. Three administrative classes volunteered: science-oriented: 60; education: 62; arts: 53, yielding a total class enrolment of 175 students. All three classes participated in 3H-integrated activities for the full semester and provided consent for anonymized data use.

Although course enrolment was compulsory, research participation (i.e., consent for research data use) was voluntary. Students were informed that participation would not affect grading and that consent concerned anonymized materials and research data (e.g., observation notes and classroom artifacts).

### 3.4 Focal cases

From the participating cohort (total class enrolment = 175), 12 focal students were selected using purposive, maximum-variation sampling to enable longitudinal tracing of individual learning trajectories within whole-class teaching. Selection aimed to maximize heterogeneity across gender, disciplinary background (science-oriented, education, arts), and classroom participation profile (from relatively quiet to highly engaged). To reduce power dynamics, we did not recruit students who were directly taught or graded by any member of the research team during the same semester. Recruitment combined open invitations and faculty-secretary facilitation. Focal case profiles are provided in Supplementary Table S3.

### 3.5 Ethical considerations

The study followed principles of voluntary participation, informed consent, the right to withdraw, and no adverse consequences. The 12 focal participants received a small milk tea/coffee voucher after completing individual interviews. Reflective journals were part of routine formative assessment and contributed to continuous assessment; therefore, no additional incentives were provided for journaling. Ethical approval was obtained from the Universiti Putra Malaysia Ethics Committee (JKEUPM-2024-1045). All data were anonymized and handled under confidentiality and data-security procedures.

### 3.6 Data collection

To capture 3H-related learning processes over the 19-week semester, we used multi-source qualitative data centered on the 12 focal cases.

Observations. Structured classroom observations were conducted throughout the semester using a standardized protocol aligned with 3H activities. Rather than observing all 175 students, observations focused on the 12 focal students to document participation and engagement during key learning episodes. Notes were used mainly to contextualize and triangulate self-reports rather than as standalone outcome measures. Each class included four focal students; typically one to two researchers attended a session, with two researchers jointly tracking the four focal students in that class. Observers used a shared seating map and recorded time-stamped fieldnotes with a structured observation template aligned with pre-specified 3H indicators. More than half of sessions involved dual observers, followed by brief post-class reconciliation of key events and discrepancies. The team also held periodic calibration meetings to align observation foci and interpretive thresholds across weeks and classes.

Interviews. At semester end, each focal student completed a semi-structured interview (45–60 min) addressing perceived changes in understanding (head), affective experience and regulation (heart), and course-related actions in daily contexts (hands). Interviews were conducted after continuous assessment grades were released, and participants were reminded that interview participation and responses would not affect grading.

Reflective journals and artifacts. Reflective journals were collected as part of course formative assessment. Students were encouraged to write first-person, context-rich narratives about classroom triggers, peer interactions, and internal struggles. Focal participants’ learning products (e.g., project outputs) were also collected; these materials were not treated as primary text data for full thematic coding, but were used to supplement Hands-related evidence and integrative trajectories and to support triangulation where appropriate.

### 3.7 Data analysis

Data were analysed using thematic analysis (Braun and Clarke, 2006) through a two-stage coding process combining deductive and inductive strategies (Yin, 2014; Saldaña, 2021) across observation fieldnotes, interview transcripts, and reflective journals.

Stage 1 applied a deductive lens using 3H as an organizing scaffold (e.g., conceptual understanding, value identification, action practice), alongside open inductive coding to capture emergent concepts not fully anticipated by the framework (e.g., critical reflection, collaborative leadership). Stage 2 involved iterative refinement of the codebook through constant comparison across cases and sources (merging/splitting codes; pattern coding into higher-order categories). Inductive codes (e.g., online information verification, peer conflict mediation) were retained even when they complicated the initial 3H structure, and were used to specify enabling conditions and boundary features of the 3H process.

Rigor procedures included an audit trail (memos, codebook revisions, decision logs) and triangulation across observation, journals, and interviews. Two trained coders independently double-coded approximately one third of the dataset, resolved discrepancies through discussion, and refined code definitions. Deviant-case checks were conducted by actively searching for excerpts that did not fit dominant patterns (e.g., strong moral expression without corresponding action). An external qualitative researcher not involved in the intervention reviewed selected coded excerpts and the developing thematic map; feedback was incorporated prior to finalizing themes.

## 4 Results

The thematic analysis indicated changes reported by students across the three dimensions of head, heart, and hands. Across cases, the data also suggested patterned links among these dimensions, which we interpret as a recursive learning dynamic rather than a deterministic developmental sequence. Given the single-site qualitative design, we present these patterns as empirically grounded propositions about how cognition, affect, and action may interact within this course context.

Across the 12 core cases, the extent and configuration of reported change varied. Some students described primarily cognitive shifts (for example, moving from memorisation to case-based reasoning), others emphasised affective resonance and empathy, and a smaller subset reported concrete actions outside class that they linked to course experiences. To avoid overgeneralisation, we present representative excerpts to illustrate these different trajectories and then discuss the conditions under which more integrated head–heart–hands (3H) patterns were observed.

### 4.1 Head: deepening moral cognition

Students made notable progress in moral cognition, shifting from passively receiving abstract concepts to actively engaging in and critically constructing their moral understanding.

#### 4.1.1 From abstract principles to contextualized understanding

All data sources clearly showed a shift in students’ learning approaches, from rote memorization of ethical concepts to constructing personalized and contextualized moral reasoning. Prior to the intervention, many participants described moral education as “abstract, theoretical, and exam oriented” (e.g., SI-S3; RJ-S11). The introduction of the 3H-based course reshaped their learning experience into a more contextualized, dialogic, and inquiry-driven process.

As one student explained:

“In the past, I thought morality meant rules the teacher explained and we had to memorize. Through real-life cases and group discussions, I learned to think about what moral values mean in everyday life.” (Student 11, reflection journal).

This shift from “receiving” knowledge to “constructing” knowledge encouraged students to participate more actively in moral reasoning. Another participant reflected:

“We no longer just memorize principles. We learn to question and interpret them. The teacher does not give us answers, but gives us questions.” (Student 3, interview).

One striking example emerged from a discussion on compassion and justice that used a well-known local university case as its starting point:

“When the teacher used the a widely reported campus homicide case to discuss compassion, I was shocked. It helped me understand why kindness and empathy matter for preventing tragedies. It was no longer just theory, but real life.” (Student 1, interview).

This shift from abstract learning to contextualized moral understanding suggests that students were moving toward greater moral autonomy and more sophisticated moral reasoning, outcomes long emphasized in cognitive-developmental theories of moral development (Kohlberg, 1984; Nucci, 2001).

#### 4.1.2 Case analysis and perspective taking

Observation data showed that students’ depth of analysis in case discussions increased over time. At the beginning of the course, many moral judgments were quite simple (for example, “Bullying is wrong”), but as the semester progressed, students began to integrate social, emotional, and situational factors into their reasoning:

“After our discussion on school bullying, I realized that bullies may act out of insecurity. Understanding this does not mean excusing their behavior, but it means thinking more deeply.” (Student 12, interview).

This capacity to move beyond surface-level moral labeling reflects a shift toward perspective taking, an important marker of moral maturity (Eisenberg et al., 2016). Group work played a key role in this process. Through dialogic discussion, students learned to integrate multiple viewpoints rather than cling to their initial opinions.

“Listening to different opinions forced me to reflect. I stopped insisting that I was always right and started asking why others think differently.” (Student 7, interview).

These moments illustrate that the “head” dimension of the 3H model was cultivated through active, dialogic cognitive engagement rather than one-way transmission, thereby fostering more nuanced moral reasoning.

#### 4.1.3 The emergence of critical moral reflection

Notably, the data indicated the emergence of critical moral reflection among students. They were no longer simply “learning morality,” but began to question and re-evaluate moral norms. One student described in her journal how she learned to verify moral claims encountered online:

“While making a moral-awareness video, I learned to fact-check information on the internet instead of blindly reposting fake ‘moral outrage’.” (Student 11, reflection journal).

Other students extended this reflective stance to their life planning, suggesting a higher level of moral self-awareness:

“Our discussions about life goals made me ask myself: What kind of person do I want to become? It is not only about learning values, but about living them.” (Student 3, interview).

These accounts signal the emergence of critical moral agency, in which students develop the capacity to examine, evaluate, and reconstruct their moral beliefs rather than uncritically accepting existing norms.

### 4.2 Heart: cultivating moral affect

In the “Heart” (affective) dimension, the intervention fostered strong emotional identification with core values, expanded students’ empathy, and enhanced their capacities for emotional expression and regulation.

#### 4.2.1 Value identification and sense of belonging

The affective elements of the 3H model supported students’ emotional connection with moral values, helping abstract ideals become personally meaningful commitments. In students’ narratives, this was sometimes expressed as an increased sense of belonging and civic solidarity within the university and broader community. For example, one student described how classroom discussions about “a shared community” reduced feelings of social distance and enabled more confident participation:

“At first, I worried that I would not fit into university life, but after learning about the idea of ‘one big Chinese family,’ I felt that I truly belong here. Sharing my hometown culture and food with classmates made me proud of my identity.” (Student 1, interview).

We do not treat national identity as moral maturation per se; rather, we interpret these accounts as civic/community-oriented belonging that may support inclusive care and prosocial responsibility. In this context, sharing food functioned as a situated practice of inclusion and mutual recognition, which can strengthen willingness to uphold shared norms and care for others.

Similarly, emotionally powerful stories about real-life heroes, such as the rescuers in the 2008 Wenchuan earthquake, strongly evoked students’ compassion and sense of responsibility:

“Watching the rescue stories made me realize how selfless people can be. It awakened my admiration and made me feel that I should contribute to others, even in small ways.” (Student 2, journal).

These findings align with Hoffman’s (2001) theory of empathy arousal, which suggests that moral emotions serve as a motivational bridge between moral knowledge and moral behavior.

#### 4.2.2 Empathy and affective expansion

Role-play and narrative activities embedded in the 3H intervention broadened students’ empathic imagination. Many reported that they “felt the pain of others” and understood perspectives they had never considered before:

“I played a victim of school bullying in a short film. For the first time, I felt the fear and helplessness of being bullied. It made me realize how much courage it takes to speak up. It changed how I look at others.” (Student 11, interview).

These immersive experiences facilitated affective perspective taking, enabling students to experience moral emotions through situational imagination, consistent with Dewey’s view that learning becomes more meaningful when it involves lived emotional experience.

In another case, a student who played a bystander who eventually stepped forward to defend a victim felt a clear growth in his moral courage:

“When I played a bystander who finally stood up for justice, I felt my moral courage growing. Now I feel that I should do the same in real life.” (Student 4, interview).

Here, empathy moved beyond simple emotional resonance to become reflective compassion: students not only sensed others’ feelings but also remained critically aware of their own moral agency. Through these immersive exercises, their empathy deepened (more nuanced understanding of others’ emotions) and widened (extending care to previously unfamiliar or marginalized groups). This trajectory resonates with Noddings’s (2002) view of moral education as a relational, responsive process.

#### 4.2.3 Emotion regulation and interpersonal harmony

The intervention also appeared to strengthen students’ emotion regulation and constructive communication in collaborative tasks. We treat these capacities as enabling skills that support moral agency in peer interaction (for example, managing conflict fairly and respectfully), rather than as moral outcomes in isolation. One student described learning to mediate disagreements during a team project:

“To complete our group’s moral micro-video, I had to mediate conflicts. It forced me to speak up and to listen to both sides. This made me more confident and more empathetic.” (Student 8, interview).

This maturation in emotional functioning extended beyond individual expression to the creation of a cooperative moral climate:

“At first, I was afraid to speak in class, but the teacher’s encouragement and my team’s support made me more confident. Now I feel the classroom is a community, not an exam.” (Student 3, journal).

Taken together, these accounts suggest that moral affect and domain-general social and emotional skills developed in parallel. In this study, such skills are interpreted as “supportive conditions” that help students translate moral understanding and value identification into practical, other-regarding action, especially in group-based learning and campus life (Nucci and Ilten-Gee, 2021).

### 4.3 Hands: transforming moral behavior

In the “hands” (behavioral) dimension, the course increased students’ active engagement, improved the quality and creativity of their moral actions, and fostered a growing sense of responsibility and leadership.

#### 4.3.1 Active participation and behavioral persistence

Students showed higher levels of initiative in moral practice activities, and many translated course learning into ongoing moral behaviors:

“All of us joined the moral micro-video project. No one was absent. Running around together and laughing during filming made us feel united.” (Student 1, interview).

More importantly, behavioral changes extended beyond the classroom. One student described how she developed a prosocial habit of daily good deeds:

“I used to ignore litter on the ground, but now I collect recyclable items and give them to an elderly person who sells waste. Doing one good thing every day has become my routine.” (Student 2, journal).

These cases suggest that students were gradually internalizing their moral motivations. This trend aligns with Bandura’s social-cognitive account of moral behavior, in which moral conduct moves from externally regulated compliance to internally endorsed commitment (Bandura, 1991).

#### 4.3.2 Creativity and problem-solving

The data further indicated that students’ gains in the “Hands” dimension went beyond simple participation. They began to address real moral situations in creative and systematic ways. For example, when discussing the problem of bicycles being left in disorganized ways around campus, students did not stop at complaints or punitive suggestions. Instead, they proposed strategies that combined rules with affective appeals, such as designing signs that evoke empathy and integrating “shared spaces” as a value theme in moral discussions, so that internalized recognition would feed back into everyday rule following:

“I suggested using signs that evoke empathy and embedding the norms in moral discussions, so that everyone realizes ‘other people also need space’.” (Student 9, interview).

At the skill level, students moved from being “technical operators” to “creative authors.” In the moral micro-video project, they were required to integrate narrative design, filming, and collaborative division of labor, which activated transferable practical abilities:

“I rotated between being director, camera operator, actor, and editor. I found that my abilities in creating moral stories and in post-production were better than I expected.” (Student 8, journal).

Taken together, these accounts illustrate integrated learning in which students’ moral actions were supported by the development of enabling competencies (e.g., communication, coordination, media production, and project management). Importantly, such competencies are not treated here as moral competencies in themselves; rather, they functioned as practical conditions that strengthened students’ sense of self-efficacy and their capacity to enact and communicate moral commitments in public-facing ways.

#### 4.3.3 Responsibility and leadership

Responsibility and leadership emerged as recurring themes in students’ accounts. Several students voluntarily took on organizing and facilitative roles during group tasks, which supported prosocial coordination and accountability within the learning community:

“When no one wanted to speak, I volunteered to take the lead. It taught me how to manage a team and take responsibility.” (Student 5, interview).

Acts of civic responsibility also appeared in some narratives:

“During a campus activity, I noticed a damaged national flag and kept it safe until it could be replaced. It felt like showing respect for the community we belong to.” (Student 9, interview).

We interpret such accounts as expressions of civic moral identity and community-oriented responsibility rather than as political endorsement. In this study, “national” symbols are discussed only insofar as they functioned as shared moral-cultural references that enabled students to articulate respect, public-mindedness, and a sense of duty toward others in a broader community.

It is noteworthy that many female students described experiences of empowerment through moral action:

“In our discussion on gender equality, I realized that women can also lead change. In the future, I want to work in advocacy for gender equality.” (Student 3, interview).

These findings highlight that moral behavior developed as an evolving continuum, ranging from participation in specific situations to self-driven leadership and longer-term moral aspirations.

### 4.4 Integrative effects: the spiral feedback loop in practice

Across many focal cases, students’ accounts suggested that changes in moral cognition (head), moral affect (heart), and moral action orientation (hands) were experienced as interconnected rather than strictly sequential. Given the qualitative and context-dependent nature of the evidence, we do not claim to have “validated” a causal mechanism. Instead, the multi-source data provide illustrative evidence of a recursive learning pattern in which reasoning-focused dialogue, affective resonance, and action-oriented tasks could reinforce one another over time.

For example, Student 1 described how classroom discussion around the idea of a “shared civic community” (head) helped her reinterpret an initial sense of anxiety and gradually transform it into stronger feelings of belonging and acceptance (heart). This affective shift, in turn, was associated with a small but meaningful action episode in which she shared food and cultural traditions from her hometown with classmates (Hands). She further reflected that this experience prompted continued consideration of community-oriented values and personal responsibility (returning to head).

Similarly, Student 11 described a strong emotional impact during a context-rich perspective-taking activity embedded in a group project (heart), which she associated with a deeper understanding of fairness- and justice-related issues (head). In her interview, she further reported becoming more willing to take supportive or intervention-oriented actions in everyday contexts (hands), for instance, seeking help from teachers or reporting concerns when noticing signs of bullying.

In another case, Student 8’s participation in producing a morality-themed micro-video functioned primarily as a platform for action expression and public moral communication, rather than as evidence that media production skills themselves constitute moral competence. The production process required the group to translate moral claims into messages for an audience, negotiate responsibilities with peers, and reflect on how moral narratives are constructed and justified. Student 8 linked this experience to increased action-related self-efficacy and engagement (heart), alongside further reflection on personal strengths and responsibilities (head), thereby illustrating coordination across 3H domains within an integrated learning episode.

**Figure 3:**
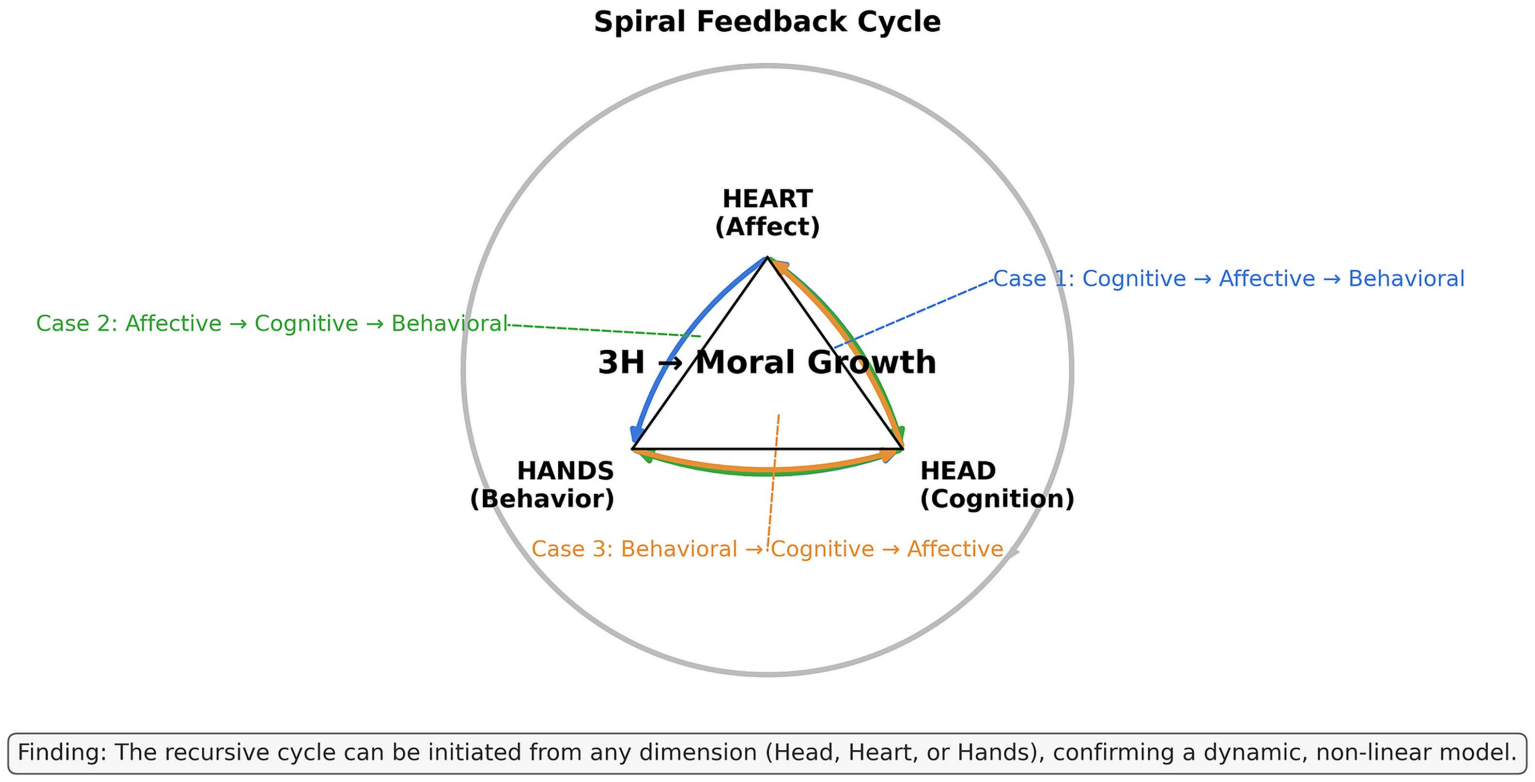
An empirically informed illustration of the 3H recursive cycle.

Taken together, these cases suggest a plausible loop in which action experiences provided material for reflection, reflection deepened cognitive understanding and sustained affective engagement, and updated cognition and affect could motivate continued participation in practice. This recursive pattern did not appear to be initiated by a single domain in a fixed order, and its strength varied across individuals and situations; however, it was recurrently described across several focal cases in ways consistent with a 3H-integrative learning pathway (see Figure 3).

### 4.5 Cross-case variation

Evidence of change was not uniform across the 12 focal cases. Head-related shifts (e.g., more contextualized conceptual understanding and more nuanced moral reasoning) were comparatively stable across cases, whereas hands-related enactments showed greater variability in intensity, persistence, and observability (Table 2). This heterogeneity may reflect differences in role allocation within group projects, baseline participation profiles (from relatively quiet to highly engaged), and variation in opportunities and constraints for transferring course-related commitments into daily contexts. These factors are treated as contextual interpretive cues rather than causal explanations.

**Table 2:** Evidence distribution matrix across the 12 focal cases.

Discipline	ID	Head	Heart	Hands	Integration	Primary sources
Science and engineering	S2	High	Medium	Medium	Medium	SI; RJ; OBS
Science and engineering	S10	Medium	Low	Medium	Medium	SI; RJ; OBS
Science and engineering	S11	Low	Medium	Medium	High	SI; RJ; OBS
Science and engineering	S12	High	Low	High	High	SI; RJ; OBS
Education	S1	Low	High	Low	Medium	SI; RJ; OBS
Education	S3	High	Low	Medium	Medium	SI; RJ; OBS
Education	S4	Medium	Low	Medium	Medium	SI; RJ; OBS
Education	S5	Medium	Medium	High	High	SI; RJ; OBS
Arts	S6	Medium	Low	High	High	SI; RJ; OBS
Arts	S7	High	Medium	Low	Medium	SI; RJ; OBS
Arts	S8	Low	Medium	Medium	Medium	SI; RJ; OBS
Arts	S9	Low	Medium	Medium	High	SI; RJ; OBS

Levels (high/medium/low) indicate relative strength of evidence within each dimension based on triangulated interpretation of OBS, RJ, and SI. “Integration” reflects the clarity of recursive linkages across head, heart, and hands. ART materials were generated as supporting instructional products (e.g., scripts or peer-review forms) and were used only for contextualization and, where relevant, triangulation, but were not coded as an independent primary dataset.

Accordingly, we interpret the 3H cycle as a situated process model whose expression and strength can differ across learners and settings, rather than as a uniform developmental outcome.

## 5 Discussion

Drawing on a semester-long intervention embedded in a Chinese university moral education course, this study offers an empirically informed account of how a head–heart–hands (3H) design can be enacted in practice and how students describe their learning experiences within that design. The findings suggest that students reported shifts in moral cognition, moral affect, and action-oriented engagement, and that the data contained recurring linkages among these dimensions that resemble a recursive learning dynamic. We interpret these patterns cautiously as qualitative evidence of a plausible process mechanism, rather than as definitive proof of effectiveness or causality.

### 5.1 Theoretical contributions: from static integration to a dynamic process mechanism

The 3H model overlaps with well-established tripartite formulations in moral and character education, such as Lickona’s (1991) knowing–feeling–doing framework and neo-Aristotelian accounts that integrate practical wisdom (phronesis), moral emotions, and habituated action (Kristjánsson, 2015; McLoughlin et al., 2025). Accordingly, we do not present 3H as a wholly novel set of components. Consistent with the process account outlined in the Literature Review (Figure 2), the contribution of this study lies in specifying integration as an instructional design principle and tracing how students narrated (and how multi-source data corroborated) transitions among cognition, affect, and action across a semester. The data suggest that concept clarification, affective engagement, and action attempts could be linked through iterative reflection and social feedback, although the strength of these linkages varied across cases and was contingent on classroom conditions.

In this way, the study translates Dewey’s proposition that learning involves the reconstruction of experience into a classroom-level account that attends to affective triggers and action-based feedback. At the same time, we acknowledge critiques of integrationist and affect-driven approaches, including concerns about over-simplifying moral development, conflating emotional arousal with moral commitment, or inadvertently encouraging performative compliance. These concerns highlight the importance of discussing boundary conditions and of treating the 3H cycle as a heuristic for design and analysis rather than a universal developmental law.

Finally, the study extends the application of 3H beyond its earlier use in ecological literacy and sustainability education (Orr, 1991; Sipos et al., 2008) to moral learning topics that students encountered in this course, such as civic responsibility, information ethics, and peer relations. This indicates that 3H may serve as a meta-framework for aligning content with learning processes, though further research across settings is needed before strong claims about transferability can be made.

### 5.2 Practical implications, cultural tensions, and critical reflections

At the practical level, the 3H framework can be read as a set of design heuristics for constructing learning experiences that connect case-based reasoning and critical discussion (head), emotionally engaging experiences (heart), and opportunities for socially oriented practice (hands). In this study, such integration appeared to help students link what they learned to how they felt and what they attempted to do. We therefore propose (rather than assert) that process-sensitive assessment approaches may be better aligned with 3H pedagogy than assessments focused solely on readily quantifiable outcomes (Biesta, 2015a).

The findings also invite reflection on how the 3H process is shaped by cultural and institutional context. In this course, the 3H design was implemented in ways that could resonate with locally meaningful concepts (for example, the unity of knowing and doing, 知行合一) and with the officially prescribed moral education curriculum (Fengyan, 2004). Rather than claiming universal “cultural adaptability” on the basis of one setting, we treat these observations as context-sensitive indications of how a general process framework may be enacted through culturally situated narratives and practices.

In our data, the “heart” dimension was often activated through shared stories, collective memories, and moral exemplars that are salient in this educational context. Such narratives appeared to generate affective engagement that, for some students, supported willingness to participate in group-based and community-oriented actions. Future comparative work is needed to examine how different cultural scripts and institutional expectations influence which affective triggers become salient and how they connect to action.

For cross-context applications, it may be useful to separate transferable structure from locally rooted content. Educators should also be attentive to the ethical risks of affect-driven pedagogy (for example, emotional manipulation or performative compliance) and preserve space for critical reflection and student agency in moral meaning-making.

Implementation also revealed practical tensions common to large compulsory courses. Uneven participation and the need for emotional safety (Zembylas, 2007) were salient, and some learning outcomes associated with heart and hands are harder to capture with conventional assessment formats (Biesta, 2015b). These tensions do not, by themselves, warrant broad institutional claims; rather, they highlight questions about how course design, assessment practices, and classroom climate can better support integrative moral learning.

### 5.3 Limitations and directions for future research

Several limitations of this study point to important directions for future work. First, the use of a single institutional site and a one-semester intervention constrains transferability and limits our ability to comment on longer-term effects. Second, participation in the 3H teaching experience occurred at the class level: three intact administrative classes volunteered from a larger first-year cohort. This self-selection may have introduced selection bias and should be considered when interpreting the findings. Third, the dataset relied heavily on reflective journals and interviews, which may be shaped by social desirability, especially in a compulsory course with an ideologically embedded curriculum and graded continuous assessment. Normative expectations in such a setting may influence students’ moral expression and value talk, particularly for affective themes such as belonging and identity. To minimise power imbalance, the study did not recruit students who were directly taught or graded by research team members in the same semester; recruitment was facilitated through open announcements and the faculty secretary, participation was voluntary, and interviews were conducted after continuous assessment grades had been released. Focal participants received small milk tea or coffee vouchers as a token of appreciation for interview time, whereas journals were a routine graded assignment for all students and did not receive additional incentives. Nevertheless, future studies could incorporate independent behavioral indicators, alternative interviewers not connected to the course, and longitudinal follow-up to better examine sustained change.

Building on these limitations, at least three lines of future research appear promising. First, longitudinal or comparative multi-site studies could examine how institutional, cultural, and curricular conditions shape students’ moral expression and the salience of affective triggers (e.g., belonging/identity), and whether the recursive linkages among head, heart, and hands emerge similarly across contexts. Second, future work could build on existing scholarship on serious moral games and game-based ethics learning (e.g., Schrier, 2015, 2017) to explore how virtual reality (VR) role play, interactive moral simulations, or AI-enabled feedback might create immersive dilemmas and support structured reflection. Related evidence from research integrity education suggests that experiential approaches with emotional involvement can be particularly effective for ethical learning outcomes (Katsarov et al., 2022). Such tools may strengthen opportunities for iteration in the head–heart–hands (3H) sequence and provide more timely and specific feedback. Third, rather than implying policy reform from the present dataset, future research could examine how assessment practices, workload structures, and professional learning communities shape the feasibility and quality of 3H-informed pedagogy in large required courses, and what forms of institutional support are most conducive to sustaining process-oriented designs.

## 6 Conclusion

In response to contemporary value challenges associated with globalization and digital media, moral education may benefit from learning designs that help students connect what they understand, feel, and attempt to do. Within the limits of this single-site qualitative study, our findings suggest that a head–heart–hands (3H)–informed instructional design can support students in linking moral concepts, affective engagement, and action-oriented attempts in a required undergraduate moral education course. Across cases, students’ accounts also showed recurring linkages among cognition, affect, and action that we interpret cautiously as a plausible recursive learning dynamic.

The study contributes by offering an empirically grounded, classroom-level process account of how integration may unfold over time when activities are intentionally aligned across head, heart, and hands. At the same time, the evidence is context-specific and primarily based on self-reports and semi-structured interviews; claims about behavioral transformation or longer-term effects therefore require longitudinal and multi-method corroboration.

Our findings also highlight contextual conditions that may shape how 3H pedagogy is enacted, including large-class formats, assessment pressures, and the need for emotionally safe participation. Moreover, because the intervention was embedded in an institutionally and ideologically normed curriculum, students’ moral expression and value talk may be shaped by normative expectations, which should be considered when interpreting affective themes such as belonging and identity.

Future research could test the robustness of the proposed recursive dynamic across different courses, institutions, and cultural–institutional contexts, including settings with different normative expectations and assessment regimes. Technology-supported formats (e.g., AI interactive moral narratives, serious moral games, or immersive simulations) may also offer promising ways to strengthen reflection and feedback while preserving student agency, but these possibilities require careful design and empirical evaluation.

Ultimately, the 3H framework highlights a practical educational aim: supporting students to reason carefully about moral questions, develop concern for others, and translate commitments into responsible action through iterative learning experiences that engage the head, heart, and hands.

## Data Availability

The original contributions presented in the study are included in the article/Supplementary material, further inquiries can be directed to the corresponding author.
