# Supplementary material for: An integrative head–heart–hands model of moral education: evidence from Chinese higher education
Source: Front Psychol. 2026 Feb 23;17:1762483. doi: 10.3389/fpsyg.2026.1762483 (PMC12968217; doi:10.3389/fpsyg.2026.1762483)
Supplement: Supplementary file 1 [file Table_1.docx]

**Supplementary Table S1. Semester-wide overview of 3H unit packages mapped to course themes (19 weeks)**

| **Course unit (weeks)** | **Core theme/value** | **Head (Moral Cognition): knowledge-building activities** | **Heart (Moral Affect): resonance-building activities** | **Hands (Moral Behavior): practice-oriented activities** |
| --- | --- | --- | --- | --- |
| Unit 1 (Weeks 1-3) | Integrity | Concept instruction on integrity and its social function; case analysis of local business integrity practices (e.g., Pangdonglai). | Reflective writing on personal trust experiences; dilemma discussion about academic misconduct and internal conflict. | Group ‘micro-initiative’ for academic integrity (e.g., honesty corner); individual academic-integrity action plan. |
| Unit 2 (Weeks 4-6) | Patriotism and national identity (community belonging/value identification) | Historical analysis of the ‘Chinese national community’ concept; discussion of forms and spirit of patriotism across periods. | ‘Hometown stories’ sharing to build cross-regional understanding; documentary viewing and discussion (e.g., earthquake relief) to explore collectivism. | Collaborative micro-film on ‘People around me’; field visit/interview (e.g., local memorial or community figure) with reflection. |
| Unit 3 (Weeks 7-10) | Justice and fairness | Introduce distributive and procedural justice; critical analysis of bullying cases (power relations, responsibility, legal boundaries). | Structured role-play (bully, victim, bystander, intervener) with guided empathy reflection (‘If I were them’). | Design and conduct a survey on perceived fairness on campus; develop a proposal to improve campus interpersonal climate. |
| Unit 4 (Weeks 11-14) | Responsibility and dedication | Link personal development to social needs; critique value deficits in ‘refined egoism’ phenomena. | ‘Ordinary heroes’ storytelling; guided reflection after service learning to deepen moral concern and value commitment. | Sustained community service with a partner organization; design a public-welfare project (e.g., addressing older adults’ digital divide). |
| Unit 5 (Weeks 15-17) | Rule of law and rule awareness | Legal principles and case discussion (e.g., cyberbullying, food safety); analyze intersections of law and morality. | Mock hearing/court to experience procedural vs substantive justice; debate ‘order relies more on law or morality’. | Campus legal literacy outreach (brochures/handbook); co-construct class rules through democratic procedures and care-oriented principles. |
| Unit 6 (Weeks 18-19) | Integration and synthesis | Review the 3H learning trajectory; write an individual moral growth analysis report. | Closing sharing ritual; screening and collective viewing of micro-film products. | Public showcase of group outputs (micro-films, survey reports, public-welfare plans); personal one-year action plan for continued practice. |

*Note. This table documents the instructional design across units. Thematic analysis was conducted primarily using OBS, RJ, and SI .*

**Supplementary Table S2. Example of an integrated 3H instructional unit (Integrity, 3 weeks)**

| **Instructional Phase / Week** | **Core Objective & 3H Focus** | **Detailed Instructional Activities** | **Time Allocation & Format** | **Data Generated (Evidence Sources)** |
| --- | --- | --- | --- | --- |
| Week 1: Cognitive Deconstruction (Head) | Objective: To understand the ethical connotation, social function, and position of "integrity" within the Socialist Core Values. Focus: Conceptual analysis, rational deliberation, case evaluation. | 1. Theoretical Lecture & Discussion: Instructor elaborates on integrity as both a moral norm and a legal principle, analyzing ethical boundaries (e.g., "white lies"). 2. Case Study (Pangdonglai Supermarket): Student groups analyze how the enterprise "Pangdonglai" translates its corporate culture of "Freedom and Love" into concrete, integrity-driven business practices (e.g., after-sales policies, employee treatment), exploring the societal value foundation of its success. | Total 90 min ▪ Lecture & Guidance: 30 min ▪ Group Case Analysis & Presentation: 60 min | Primary: OBS; Supporting: in-class group discussion notes / presentations (ART). |
| Week 2: Affective Identification (Heart) | Objective: To foster internal identification and emotional resonance with the value of integrity, and to experience the emotional consequences of dishonest behavior. Focus: Empathic experience, value sensing, emotional connection. | 1. Scenario-Based Role-Play (embedded in group work; roles are self-assigned): Groups develop short scripts for integrity-related moral dilemmas (e.g., academic plagiarism or breach of contract). Roles are allocated within groups based on students’ preferences and strengths. Students may enact stakeholders (e.g., perpetrator, victim, bystander), while others contribute as director, scriptwriter, camera operator, editor, or post-production coordinator. This design ensures that all students participate, although not everyone plays the same role. 2. Affective Reflection Circle: Structured, facilitator-led sharing session post role-play, focusing on questions like: "How did you feel when playing the victim?" "What emotional or practical difficulties must one overcome to uphold integrity?" | Total 90 min ▪ Role-play Preparation & Enactment: 50 min ▪ Affective Reflection & Sharing: 40 min | Primary: OBS; Supporting: role-play scripts / micro-film drafts and related instructional products (ART); classroom sharing notes. |
| Week 3: Practical Application & Reflection  (Hands → Head) | Objective: To translate cognitive understanding and affective resonance into preliminary practical commitments or plans, and to deepen comprehension through reflection. Focus: Action design, collaborative inquiry, reflective integration. | 1. Practical Project Design & Launch: Based on prior learning, each group develops a proposal for a feasible "micro-integrity" initiative on campus (e.g., an online "Exam Integrity Pledge" campaign, establishing an "Honesty Lost-and-Found Station"). 2. Peer Review & Proposal Refinement: Groups critique each other's proposals, focusing on feasibility, innovation, and connection to the 3H learning process. 3. Unit Integration Reflection: Students submit individual reflective journals, systematically reviewing the entire process from cognitive case study (Head), affective experience (Heart), to action design (Hands), explaining their deepened understanding. | Total 90 min ▪ Group Project Design & Development: 40 min ▪ Peer Review & Discussion: 30 min ▪ Instructor Summary & Reflection Guidance: 20 min | Primary: RJ; OBS; SI. Supporting: peer-review forms/proposals and revised products (ART). |

*Note. OBS = classroom observation notes; RJ = reflective journals; SI = semi-structured interviews; ART = supporting instructional products (e.g., scripts or peer-review forms). Thematic analysis was conducted primarily using OBS, RJ, and SI; ART materials were used only for contextualization and, where relevant, triangulation, but were not coded as an independent primary dataset.*

**Supplementary Table S3. Profiles of the 12 focal cases and sampling characteristics**

| **Discipline** | **ID** | **Gender** | **Age** | **Background** | **Classroom participation** | **Key Features of Participation** |
| --- | --- | --- | --- | --- | --- | --- |
| Science & Engineering | S2 | F | 19 | Urban | relatively silent | Skilled in media production; evidence-based reasoning. |
| Science & Engineering | S10 | M | 18–19 | Rural | moderate participation | Strong in research and editing; innovative problem-solver. |
| Science & Engineering | S11 | F | 18 | Rural | moderate participation | Empathy through role-play; moral action intention. |
| Science & Engineering | S12 | M | 19 | Rural | active participation | Overcame teamwork issues; applied theory in practice. |
| Education | S1 | F | 18 | Rural, | moderate participation | Developed cultural confidence; promotes empathy via storytelling. |
| Education | S3 | F | 19 | Urban | relatively silent | Reflective learner; values inquiry over memorization. |
| Education | S4 | M | 18 | Rural | active participation | Proposes practical solutions; active in group work. |
| Education | S5 | M | 19 | Urban | highly engaged | Student leader; improved self-efficacy through projects. |
| Arts | S6 | F | 20 | Urban | highly engaged | Sustained moral behavior; active in volunteering. |
| Arts | S7 | F | 19 | Urban | highly engaged | Advocates formative assessment; expressive and reflective. |
| Arts | S8 | M | 18–19 | Urban | moderate participation | From introverted to proactive; creative participation. |
| Arts | S9 | M | 19 | Urban | highly engaged | Strong civic responsibility; connects learning to action. |

Note. The 12 focal students were selected using maximum-variation purposive sampling across discipline, gender, background, and classroom engagement. Core participants received small milk tea/coffee vouchers as a token of appreciation. Reflective journals were part of continuous assessment. Semi-structured interviews were conducted after continuous assessment grades were released; research participation was non-evaluative and did not affect course evaluation.
